# A Longitudinal Study of the Antioxidant Barrier and Oxidative Stress in Morbidly Obese Patients after Bariatric Surgery. Does the Metabolic Syndrome Affect the Redox Homeostasis of Obese People?

**DOI:** 10.3390/jcm9040976

**Published:** 2020-04-01

**Authors:** Barbara Choromańska, Piotr Myśliwiec, Magdalena Łuba, Piotr Wojskowicz, Jacek Dadan, Hanna Myśliwiec, Katarzyna Choromańska, Anna Zalewska, Mateusz Maciejczyk

**Affiliations:** 1 Department of General and Endocrine Surgery, Medical University of Bialystok, 24a M. Sklodowskiej-Curie Street, 15-276 Bialystok, Poland; piotr.a.mysliwiec@gmail.com (P.M.); ananau@wp.pl (M.Ł.); pwojsk@wp.pl (P.W.); jacdad@poczta.onet.pl (J.D.); 2 Department of Dermatology and Venereology, Medical University of Bialystok, 14 Żurawia Street, 15-540 Bialystok, Poland; hanna.mysliwiec@gmail.com; 3 Department of Oral Surgery, Medical University of Gdansk, 7 Dębinki Street, 80-211 Gdansk, Poland; kasia24_89@o2.pl; 4 Experimental Dentistry Laboratory, Medical University of Bialystok, 24a M. Sklodowskiej-Curie Street, 15-274 Bialystok, Poland; azalewska426@gmail.com; 5 Department of Hygiene, Epidemiology and Ergonomics, Medical University of Bialystok, 2c Mickiewicza Street, 15-233 Bialystok, Poland

**Keywords:** oxidative stress, redox biomarkers, morbid obesity, bariatric surgery

## Abstract

This is the first study to evaluate both the antioxidant barrier, glutathione metabolism, and oxidative damage to proteins and lipids in morbidly obese patients undergoing bariatric treatment. The study included 65 patients with class 3 obesity divided into two subgroups: morbidly obese patients without metabolic syndrome (OB) and obese patients with metabolic syndrome (OB + MS). Blood samples were collected before surgery as well as one, three, six, and twelve months after the bariatric treatment. Superoxide dismutase and reduced glutathione (GSH) were significantly decreased, whereas glutathione reductase and uric acid were enhanced in morbidly obese patients before bariatric surgery as compared to lean control. Moreover, in the OB group, we observed the increase of superoxide dismutase (SOD) and the decrease of uric acid (UA) after the bariatric treatment; however, these changes were not observed in the OB + MS group. The oxidative damage to proteins (advanced glycation end products, AGE; advanced oxidation protein products, AOPP) and lipids (8-isoprostanes, 8-isop; 4-hydroxynoneal) was higher in OB as well as OB + MS patients. We noticed that AGE and AOPP levels diminished after the bariatric treatment, whereas redox status (ratio of GSH to oxidized glutathione) was still reduced in the OB + MS group. Summarizing, morbid obesity is associated with disturbances in the antioxidant barrier and enhanced oxidative damage to proteins and lipids. Although bariatric surgery improves redox homeostasis in obese patients, those with metabolic syndrome show a continuous decrease in the antioxidant status. In patients undergoing bariatric treatment, antioxidant supplementation may be considered.

## 1. Introduction

Morbid obesity (body mass index (BMI) > 40 kg/m^2^) is one of the most serious health problems in the world. Many epidemiological studies have shown that obesity is frequently accompanied by metabolic disorders like hypertension, insulin resistance, type 2 diabetes (T2DM), ischemic heart disease, as well as cancer [1,2]. However, despite intensive research on morbid obesity, it remains unclear why some obese subjects develop the metabolic syndrome (MS), and some do not. It is well known that the development of obesity is caused by adipokine secretion like leptin, adipokine or resistin as well as an increased expression of fatty acid/cholesterol transporters in the target tissues [3,4,5]. Nevertheless, recent studies emphasize the critical role of oxidative stress (OS) in the development of obesity and its metabolic complications [6,7]. OS occurs when cellular components (e.g., lipids and proteins) are oxidized and cellular metabolism is disturbed. This condition is mainly due to the overproduction of reactive oxygen (ROS) and nitrogen (RNS) species [8]. The first sign of OS is lipid peroxidation because the cell membrane is the first to be exposed to ROS. Oxidative damage to lipids leads to the formation of peroxides such as 4-hydroxynonenal (4-HNE) and 8-isoprostanes (8-isoP); however, excessive formation of ROS also causes modifications of proteins and amino acid residues [9]. It was shown that protein/lipid oxidation products are very cytotoxic, leading to cell death by apoptosis and necrosis [10]. Therefore, in aerobic organisms, several antioxidant systems protect cells against OS. Antioxidants not only inhibit ROS-induced oxidation but also repair some forms of oxidative modification in biomolecules. The most important blood antioxidants include superoxide dismutase (SOD), catalase (CAT), glutathione peroxidase (GPx), glutathione reductase (GR), reduced glutathione (GSH) and uric acid (UA) [7,8,11]. It is suggested that antioxidant supplementation could reduce metabolic disorders and improve the condition of obese patients. However, little is still known about the efficiency of the antioxidant barrier in morbidly obese cases. Particularly, the unknown is the impact of metabolic syndrome on the redox homeostasis of these patients.

Nowadays, bariatric surgery is the most efficient method of obesity treatment [12]. Many studies have shown that bariatric surgery not only helps patients achieve long-term weight loss but also removes obesity-related complications, including T2DM and high blood pressure [13,14,15,16]. It has been demonstrated that weight-loss by laparoscopic sleeve gastrectomy leads to a 40%–65% reduction in excess weight. Moreover, 56% of obese patients achieve resolution of T2DM [17]. However, the exact reason for these changes is still unknown. It is postulated that the improvement of obese patients is associated with the reduction of oxidative stress levels [7,8,11]; however, there is no longitudinal data about the redox homeostasis of patients undergoing bariatric treatment. Therefore, the aim of our study was to evaluate the impact of morbid obesity as well as huge weight loss on the blood antioxidant systems/oxidative stress before as well as one, three, six and twelve months after bariatric surgery. For this purpose, we assessed the enzymatic and non-enzymatic antioxidant barrier, redox potential, as well as oxidative damage to proteins and lipids in the plasma and serum of both study groups and healthy controls. We are also the first to compare redox homeostasis in obese patients with metabolic syndrome to obese cases only.

## 2. Materials and Methods

The study was approved by the Ethics Committee of the Medical University of Bialystok (permission numbers: R-I-002/69/2012 and R-I-002/187/2017). All procedures were designed, conducted, and reported in compliance with the Declaration of Helsinki, according to the guidelines for Good Clinical Practice. All subjects gave their informed consent to participate in the study.

The study included 65 patients (women aged from 19 to 65 years) with class 3 obesity (BMI > 40 kg/m^2^), who underwent elective bariatric surgery-laparoscopic sleeve gastrectomy. Patients were treated at the First Department of General and Endocrine Surgery at the University Hospital in Bialystok. The study group was divided into two subgroups: morbidly obese patients without metabolic syndrome (OB) (*n* = 34) and morbidly obese patients with metabolic syndrome (OB + MS) (*n* = 31). Material for testing was collected before surgery (OB 0; OB + MS 0) as well as one month (OB 1; OB + MS 1), three months (OB 3; OB + MS 3), six months (OB 6; OB + MS 6) and twelve months (OB 12; OB + MS 12) after bariatric treatment.

Thirteen patients were treated for type 2 diabetes mellitus (T2DM), and twenty-two patients were treated for hypertension. Patients with obesity had a mean weight loss of 5 ± 0.6 kg for a time interval of 10 to 30 days prior to surgery. It was associated with a low-calorie diet, which was a part of the preparation for the surgery.

Body weight, height and waist and hips circumferences were measured using standard methods. Waist circumference was measured halfway between the lower arch of the ribs and the upper edge of the iliac crest, and the circumference of the hips by the largest protrusion of the gluteal muscles, below the iliac plates. BMI was calculated as weight (kg) divided by the square of height (m^2^). The metabolic syndrome (MS) was diagnosed in accordance with the International Diabetes Federation. A subject has metabolic syndrome if it satisfies three or more of the following traits: large waist circumference (at least 89 cm for women and 102 cm for men or waist circumference does not need to be measured if BMI is >30 kg/m^2^, central obesity can be assumed), hypertriglyceridemia (150 mg/dL) or specific treatment of this lipid abnormality, reduced high-density lipoprotein (HDL) cholesterol (less than 40 mg/dL in men or less than 50 mg/dL), hypertension (130/85 mm Hg or higher) or treatment of previously diagnosed hypertension and elevated fasting blood glucose (100 mg/dL or higher) or previously diagnosed type 2 diabetes [18].

The control group consisted of 33 lean healthy women (aged from 19 to 65 years; BMI < 25 kg/m^2^) attending follow-up visits at the Specialist Dental Clinic at the Medical University of Bialystok. Only patients with normal blood counts and biochemical blood tests (Na^+^, K^+^, creatinine, AST, ALT, INR) were included in the control group.

The exclusion criteria for both the study and control group comprised systemic diseases: metabolic diseases (type 1 diabetes, gout, osteoporosis, and mucopolysaccharidosis), autoimmune diseases (Hashimoto’s disease, Crohn’s disease, and ulcerative colitis), infectious diseases (hepatitis A, B, or C, HIV/AIDS), diseases of the cardiovascular (other than hypertension), respiratory, digestive (other than obstructive sleep apnea), and genitourinary systems.

Additional exclusion criteria for the control group were hypertension, insulin resistance and type 2 diabetes, and obstructive sleep apnea.

Within the three-month period preceding the study, patients and healthy controls declared not taking any antibiotics, nonsteroidal anti-inflammatory drugs, glucocorticosteroids, vitamins, and antioxidant supplements. The participants were non-smokers and did not drink alcohol more frequently than once a month. Pregnant women, patients with acute inflammatory infections and a history of malignancy were also excluded from the study.

The clinical characteristics of the control and study groups are shown in Table 1.

### 2.1. Blood Collection

All samples were collected from obese and lean patients in the overnight fasting state. Twenty-four hours before blood sampling, patients also did not practice intense physical activity. The blood was taken to serum and EDTA tubes (S-Monovette SARSTEDT). The blood samples were centrifuged for 10 min at 4000 rpm in 4 °C. The supernatant was retained for further testing. Butylated hydroxytoluene (BHT) was added to all samples to protect them against oxidation (10 μL 0.5 M BHT/1 mL serum/plasma) [19]. The samples were stored at −80 °C until final examinations.

### 2.2. Laboratory Measurements

Serum triglycerides, total cholesterol, low-density lipoprotein (LDL) and high-density lipoprotein (HDL) cholesterol, C-reactive protein (CRP), alanine transaminase (ALT), aspartate transaminase (AST), the full blood count, glucose, and insulin were quantified by using an Abbott analyzer (Abbott Diagnostics, Wiesbaden, Germany). Homeostatic model assessment (HOMA-IR = fasting glucose (mg/dl) x fasting insulin (mU/l)/405) index was calculated [20].

### 2.3. Redox Assays

For the redox assays, all reagents were obtained from Sigma-Aldrich (Nümbrecht, Germany/Saint Louis, MO, USA). Antioxidant enzymes were assessed in serum, whereas the non-enzymatic antioxidants, redox status, and oxidation products were assessed in the plasma. The absorbance/fluorescence was measured using a 96-well microplate reader Infinite M200 PRO Multimode (Tecan Group Ltd., Männedorf, Switzerland). All determinations were performed in duplicate samples and standardized to 1 mg of total protein. Total protein content was determined colorimetrically using the bicinchoninic acid assay with bovine serum albumin as a standard (Thermo Scientific PIERCE BCA Protein Assay Kit, Rockford, IL, USA).

#### 2.3.1. Antioxidant Barrier

The activity of serum Cu-Zn-superoxide dismutase (SOD, EC 1.15.1.1) was assessed spectrophotometrically by measuring the inhibition rate of adrenaline oxidation at 480 nm [21]. One unit of SOD activity was defined as the quantity of enzyme inhibiting adrenaline oxidation by 50%. The activity of serum catalase (CAT, EC 1.11.1.6) was assessed spectrophotometrically by measuring hydrogen peroxide (H_2_O_2_) decomposition at 240 nm [22]. One unit of CAT activity was defined as the quantity of the enzyme catalyzing decomposition of 1 mM of H_2_O_2_ per 1 min. The activity of serum glutathione peroxidase (GPx, EC 1.11.1.9) was assessed spectrophotometrically at 340 nm based on the reduction of organic peroxides by GPx in the presence of reduced nicotinamide adenine dinucleotide phosphate (NADPH) [23]. The activity of serum glutathione reductase (GR, EC 1.8.1.7) was assessed spectrophotometrically at 340 nm by measuring the decrease in NADPH absorbance [24]. One unit of GR activity was defined as the amount of enzyme catalyzing the oxidation of 1 μM NADPH per 1 min.

The concentration of plasma glutathione was assessed colorimetrically using the enzymatic reaction with NADPH, 5,5′-dithiobis-(2-nitrobenzoic acid) (DTNB), and GR [25,26]. The absorbance was measured at 412 nm. The reduced glutathione (GSH) concentration was calculated from the difference between the concentration of total glutathione and oxidized glutathione (GSSG). Oxidation/reduction potential (redox status) was calculated based on the formula = (GSH)^2^/(GSSG) [27].

The concentration of plasma uric acid (UA) was assessed spectrophotometrically at 630 nm using the commercial kit (QuantiChromTM Uric Acid DIUA-250; BioAssay Systems, Harward, CA, USA), according to the manufacturer’s instructions.

#### 2.3.2. Oxidative Stress Products

The content of plasma advanced glycation end products (AGE) was assessed spectrofluorimetrically by measuring AGE-specific fluorescence at 350/440 nm [28]. Immediately before the assay, plasma samples were diluted (1:5, v:v) in 0.02 M phosphate-buffered saline (PBS), pH 7.4 [29]. The concentration of plasma advanced oxidation protein products (AOPP) was assessed spectrophotometrically at 340 nm⁠ by measuring the iodide ion oxidizing capacity of the plasma [28]. Immediately before the assay, plasma was diluted (1:5, v:v) in 0.02 M PBS [29]. The concentration of plasma 4-hydroxynoneal protein adducts (4-HNE) and 8-isoprostanes (8-isop) was evaluated using commercial ELISA kits (OxiSelect HNE Adducts Competitive ELISA Kit, Cell Biolabs, Inc., San Diego, CA; 8-Isoprostane ELISA Kit, Cayman Chemicals, Ann Arbor, MI, USA; respectively), according to the manufacturer’s instructions.

#### 2.3.3. Statistical Analysis

Statistical analysis was performed using GraphPad Prism 8.3.0 for MacOS (GraphPad Software, Inc. La Jolla, USA). The normality of the distribution was assessed using the Shapiro–Wilk test. For comparison of quantitative variables, the Kruskal–Wallis ANOVA test and Dunn’s test were used. Multiplicity adjusted p-value was also calculated. The relationship between the assessed redox biomarkers was evaluated using the Spearman rank correlation. In order to determine the diagnostic utility of measured parameters, receiver operating characteristic (ROC) curves were drawn, and the area under the curve (AUC) was calculated. The statistical significance level was set at p < 0.05.

The number of subjects was determined based on our previous experiment, assuming that the power of the test would be equal to 0.9.

## 3. Results

Table 1 shows a comparison of the clinical and laboratory characteristics of the lean control (C), patients with morbid obesity without metabolic syndrome (OB 0) and patients with morbid obesity and metabolic syndrome (OB + MS 0) before and after the bariatric surgery. We found significantly higher values in BMI, waist-hip ratio (WHR), and waist circumference as well as in CRP, white blood cell, glucose, and insulin levels and HOMA-IR, in both groups of patients with obesity compared with lean patients, whereas HDL levels were decreased. Total cholesterol, low-density lipoprotein concentration, and blood pressure were greater in OB + MS 0 patients compared with controls. The body weight, BMI as well as WHR diminished after bariatric surgery in both studied groups at six and twelve months after bariatric surgery. Additionally, we noticed significant differences between OB 3 and OB + MS 3 as well as OB 6 and OB + MS 6 regarding the following: fasting plasma glucose and TAG (Table 1).

Individual data of metabolic parameters are presented in Supplementary Materials (Table S1).

### 3.1. Antioxidant Barrier

Both enzymatic (SOD, CAT, GPx, and GR) and non-enzymatic antioxidants (UA, GSH, and GSSG) were used to assess the antioxidant status. Redox potential [GSH]^2^/[GSSG] has also been calculated.

#### 3.1.1. Superoxide Dismutase (SOD)

**Figure 1:**
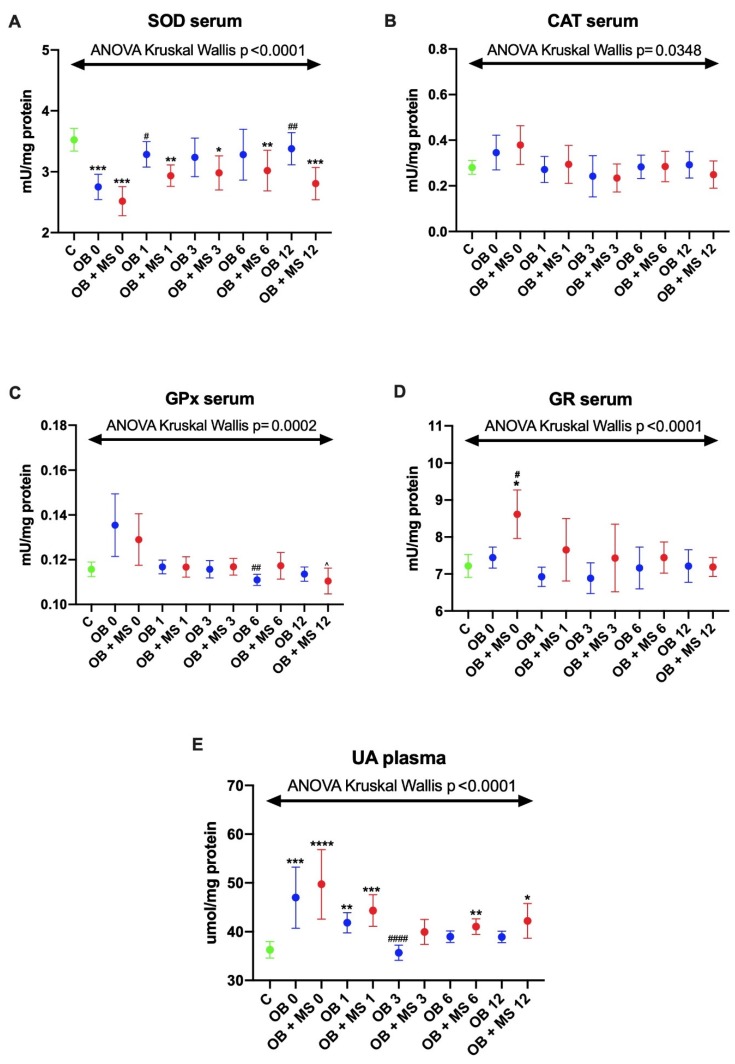
Activity of serum enzymatic (**A–D**) and plasma non-enzymatic antioxidants (**E**) of the control, morbid obesity without metabolic syndrome (OB) and morbid obesity with metabolic syndrome (OB + MS). Results are presented as median with 95% Cl. * p < 0.05, ** p < 0.01, *** p < 0.001, **** p < 0.0001 indicate significant differences from the control; # p < 0.05, ## p < 0.01, #### p < 0.0001 indicate significant differences from the morbid obesity without metabolic syndrome (OB 0) patients before bariatric surgery. Superoxide dismutase (SOD), catalase (CAT), glutathione peroxidase (GPx), glutathione reductase (GR) and uric acid (UA), morbidly obese patients without metabolic syndrome (OB) and morbidly obese patients with metabolic syndrome (OB + MS), before (OB 0; OB + MS 0) as well as one month (OB 1; OB + MS 1), three months (OB 3; OB + MS 3), six months (OB 6; OB + MS 6) and twelve months (OB 12; OB + MS 12) after bariatric surgery.

The activity of serum SOD was significantly decreased in both studied groups before the bariatric surgery: OB 0 (−23%, p < 0.0001) and OB + MS 0 (−27%, p < 0.0001) as compared to lean control. Interestingly, in morbidly obese patients with metabolic syndrome: OB + MS 1 (−19%, p = 0.0064), OB + MS 3 (−16%, p = 0.0282), OB + MS 6 (−18%, p = 0.0085) and OB + MS 12 (−21%, 0.0007), the activity of SOD was also lower in every time period after the surgery than in lean individuals, whereas we did not observe any changes in patients with morbid obesity without metabolic syndrome at the same time period. Furthermore, in OB 1 and OB 12 groups, we found the increase in SOD activity (+20%, p = 0.0295 +26%, p = 0.003, respectively) in comparison with OB 0 patients (Figure 1A).

#### 3.1.2. Catalase (CAT) and Glutathione Peroxidase (GPx)

There were no statistically significant differences in the activity of serum CAT and GPx in the serum of studied groups (OB and OB + MS) compared with control. Also, the GPx activity did not change in OB and OB + MS patients after bariatric treatment (Figure 1B–D).

#### 3.1.3. Glutathione Reductase (GR)

The activity of serum GR was significantly increased in the OB + MS 0 (+15%, p = 0.05) group as compared to healthy controls (+19%, p = 0.05) and the OB subgroup (+16%, p = 0.05).

#### 3.1.4. Uric Acid (UA)

The plasma concentration of UA was significantly higher before (OB 0: +15%, p = 0.0002) as well as one month (OB 1: +13%, p = 0.0029) after the bariatric surgery in morbid obesity without metabolic syndrome patients compared to the control group. Furthermore, we observed a decrease in UA concentration in the OB 3 group (−17%, p < 0.0001) compared with OB 0. Interestingly, in plasma of morbidly obese subjects with metabolic syndrome, the UA concentration was greater in OB + MS 0 (+20%, p < 0.0001), OB +MS 1 (+14%, p = 0.0003), OB + MS 6 (+13%, p = 0.0045) and OB + MS 12 (+10%, p = 0.0187) as compared to lean individuals (Figure 1E).

#### 3.1.5. Reduced Glutathione (GSH)

**Figure 2:**
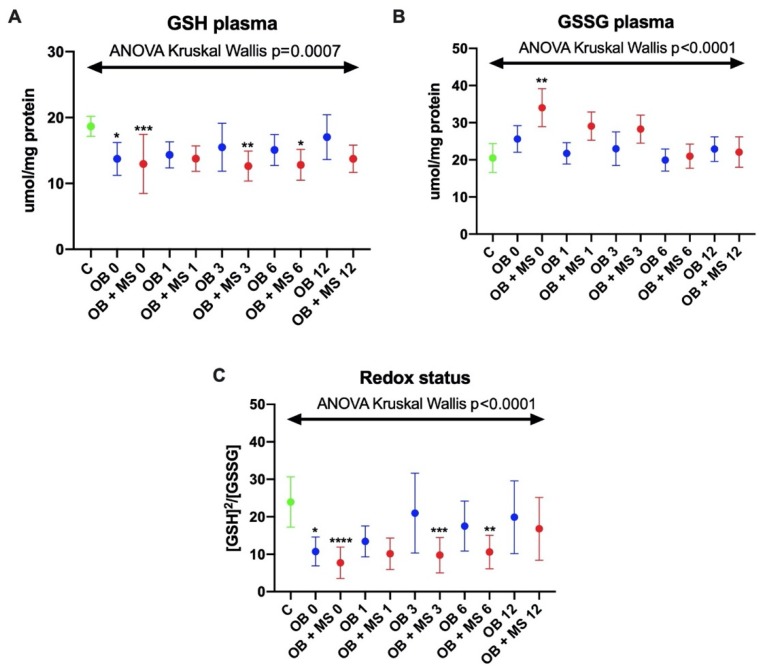
Plasma concentration of glutathione (GSH) (**A**), glutathione disulfide (GSSG) (**B**) and redox status (**C**) of the control, morbid obesity without metabolic syndrome (OB) and morbid obesity with metabolic syndrome (OB + MS). Results are presented as median with 95% Cl. *p < 0.05,** p < 0.01, *** p < 0.001, **** p < 0.0001 indicate significant differences from the control; glutathione (GSH), glutathione disulfide (GSSG), morbidly obese patients without metabolic syndrome (OB) and morbidly obese patients with metabolic syndrome (OB + MS), before (OB 0; OB + MS 0) as well as one month (OB 1; OB + MS 1), three months (OB 3; OB + MS 3), six months (OB 6; OB + MS 6) and twelve months (OB 12; OB + MS 12) after bariatric surgery.

The concentration of GSH was significantly lower in the plasma of both obese groups before the bariatric surgery: OB 0 (−26%, p = 0.0447) and OB + MS 0 (−33%, p = 0.0001) as compared to control. In addition, the GSH concentration was significantly decreased in the plasma of morbidly obese patients with metabolic syndrome in three (OB + MS 3: −31%, p = 0.0040) and six (OB + MS 6: −25%, p = 0.0108) months after bariatric treatment (Figure 2A).

#### 3.1.6. Glutathione Disulfide (GSSG)

The concentration of plasma GSSG was only markedly higher in OB + MS 0 patients (+89%, p = 0.0015) as compared to the control group (Figure 2B).

#### 3.1.7. Redox Status

Plasma redox status was significantly decreased in OB 0 (−46%, p = 0.0105) and OB + MS 0 (−82%, p < 0.0001) in comparison with the control group. Interestingly, in morbidly obese patients with metabolic syndrome it was still diminished after bariatric surgery: OB + MS 3 (−77%, p = 0.0004) and OB + MS 6 (−54%, p = 0.0088) (Figure 2C).

### 3.2. Oxidative Damage Products

Oxidative stress was assessed based on the protein (AGE, AOPP) and lipid (4-HNE, 8-isoP) oxidative damage.

#### 3.2.1. Advanced Glycation End Products (AGE)

**Figure 3:**
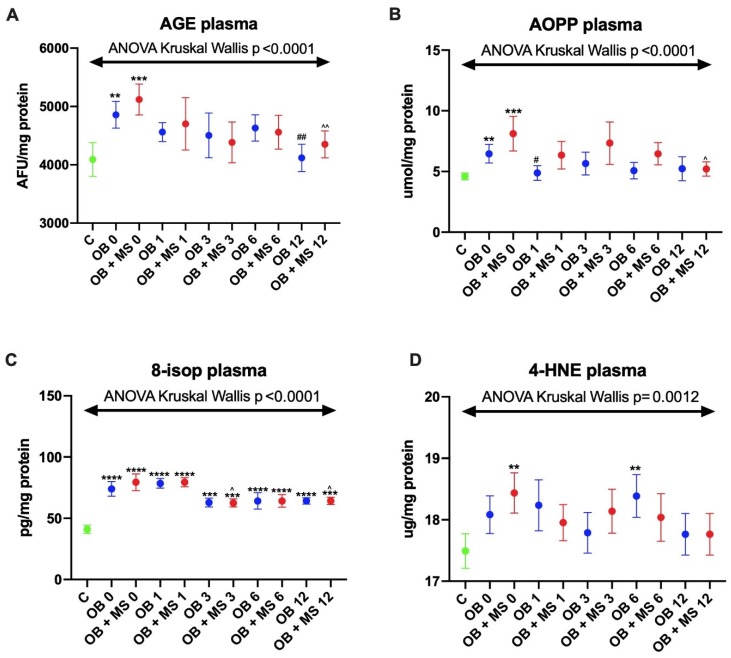
Plasma content of advanced glycation end products (AGE) (**A**), plasma concentration of advanced oxidation protein products (AOPP) (**B**), 8-isoprostanes (8-isop) (**C**) and 4-hydroxynoneal protein adducts (4-HNE) of the control (**D**), morbid obesity without metabolic syndrome (OB) and morbid obesity with metabolic syndrome (OB + MS). Results are presented as median with 95% Cl. ** p < 0.01, *** p < 0.001, **** p < 0.0001 indicate significant differences from the control; ## p < 0.01 indicate significant differences from the morbid obesity without metabolic syndrome (OB 0) patients before bariatric surgery; ^ p < 0.05, ^^ p < 0.01 indicate significant differences from the morbid obesity with metabolic syndrome (OB + MS 0) patients before bariatric surgery. Advanced glycation end products (AGE), advanced oxidation protein products (AOPP), 8-isoprostanes (8-isop) and 4-hydroxynoneal protein adducts (4-HNE), morbidly obese patients without metabolic syndrome (OB) and morbidly obese patients with metabolic syndrome (OB + MS), before (OB 0; OB + MS 0) as well as one month (OB 1; OB + MS 1), three months (OB 3; OB + MS 3), six months (OB 6; OB + MS 6) and twelve months (OB 12; OB + MS 12) after bariatric surgery.

The AGE plasma content significantly increased in morbidly obese patients without metabolic syndrome OB 0 (+23%, p = 0.0026) as well as in those with metabolic syndrome OB + MS 0 (+31%, p < 0.0001) as compared to the control group. Moreover, we noticed that, the AGE content diminished in both obese groups twelve months after bariatric treatment OB 12 (−16%, p = 0.0041 vs. OB 0) and OB + MS 12 (−17%, p = 0.0018 vs. OB + MS 0) (Figure 3A).

#### 3.2.2. Advanced Oxidation Protein Products (AOPP)

We found a markedly higher plasma concentration of AOPP in obese patients: OB 0 (+32%, p = 0.0099) and OB + MS 0 (+64%, p = 0.0001) as compared to lean ones. Similar to the AGE content, the AOPP concentration decreased after the bariatric surgery: OB 1 (−25%, p = 0.0323 vs. OB 0) and OB + MS 12 (−34%, p = 0.0417 vs. OB +MS 0) (Figure 3B).

#### 3.2.3. 8-Isoprostanes (8-isop)

The 8-isop concentration was significantly greater in both morbidly obese groups before bariatric surgery as well as in every time period after the surgery compared to lean patients: OB 0 (+82%, p < 0.0001), OB 1 (+99%, p < 0.0001), OB 3 (+46%, p = 0.0002), OB 6 (+59%, p < 0.0001), OB 12 (+48%, p < 0.0001), OB + MS 0 (+77%, p < 0.0001), OB + MS 1 (+98%, p < 0.0001), OB + MS 3 (+49%, p = 0.0002), OB + MS 6 (+48%, p < 0.0001), and OB + MS 12 (+46%, p = 0.0002). Interestingly, in morbidly obese group with metabolic syndrome, the plasma concentration of 8-isop diminished three and twelve months after the bariatric surgery: OB + MS 3 (−17%, p = 0.014 vs. OB +MS 0) and OB + MS 12 (−18%, p = 0.0194 vs. OB + MS 0) (Figure 3C).

#### 3.2.4. 4-Hydroxynoneal Protein Adducts (4-HNE)

We found differences in the plasma 4-HNE concentration of OB + MS 0 (+3%, p = 0.0053) and OB 6 (+4%, p = 0.0024) patients in comparison with the control group (Figure 3D).

#### 3.2.5. Correlations

**Figure 4:**
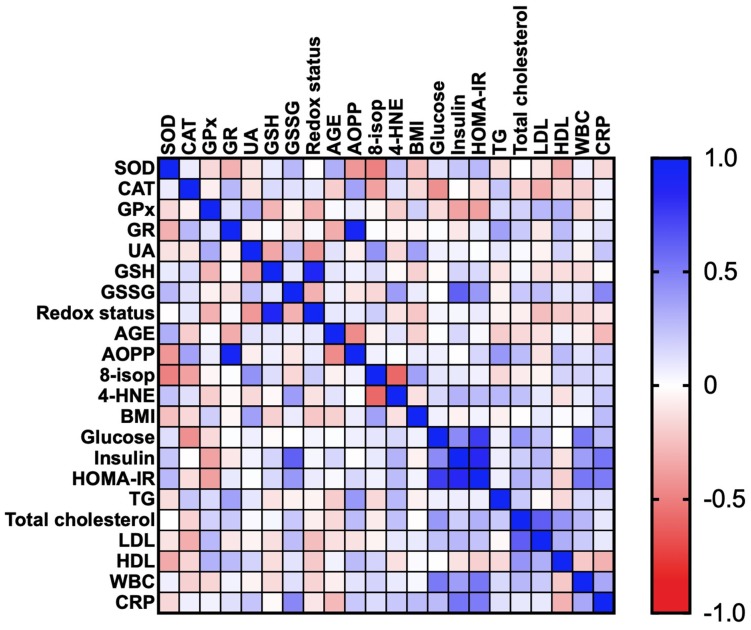
Correlations between the analyzed redox biomarkers and clinical parameters in patients with morbid obesity without metabolic syndrome (OB 0). Superoxide dismutase (SOD), catalase (CAT), glutathione peroxidase (GPx), glutathione reductase (GR) and uric acid (UA), glutathione (GSH), glutathione disulfide (GSSG) advanced glycation end products (AGE), advanced oxidation protein products (AOPP), 8-isoprostanes (8-isop) and 4-hydroxynoneal protein adducts (4-HNE), C-reactive protein (CRP), high-density lipoprotein (HDL), homeostatic model assessment of insulin resistance (HOMA-IR), low-density lipoprotein (LDL), triacylglycerol (TG), white blood cell count (WBC).

**Figure 5:**
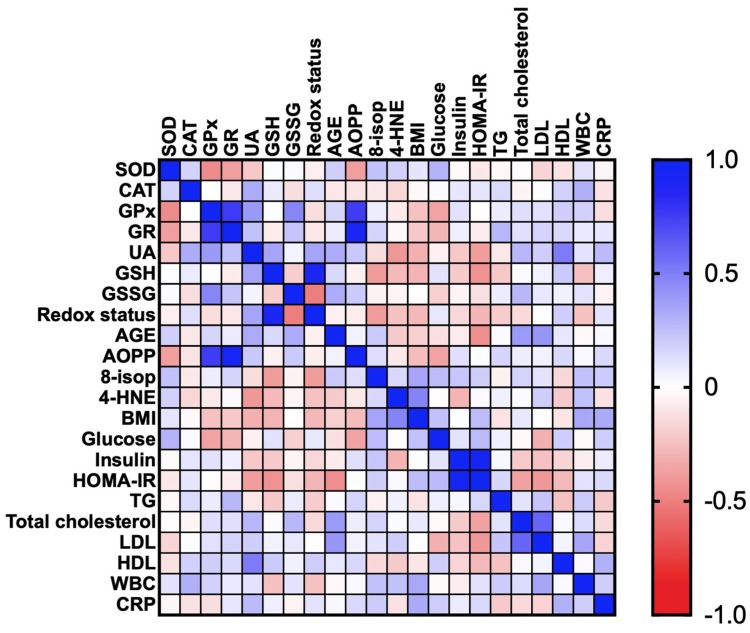
Correlations between the analyzed redox biomarkers and clinical parameters in patients with morbid obesity and metabolic syndrome (OB + MS 0). Superoxide dismutase (SOD), catalase (CAT), glutathione peroxidase (GPx), glutathione reductase (GR) and uric acid (UA), glutathione (GSH), glutathione disulfide (GSSG)advanced glycation end products (AGE), advanced oxidation protein products (AOPP), 8-isoprostanes (8-isop) and 4-hydroxynoneal protein adducts (4-HNE), C-reactive protein (CRP), high-density lipoprotein (HDL), homeostatic model assessment of insulin resistance (HOMA-IR), low-density lipoprotein (LDL), triacylglycerol (TG), white blood cell count (WBC).

Correlations between the analyzed redox biomarkers and clinical parameters are presented in the heat maps (Figure 4 and Figure 5).

In the OB subgroup, we found negative correlations between CAT and glucose (R = −0.432; p = 0.014) as well as GPx and HOMA-IR (R = −0.375; p = 0.049). Additionally, in OB patients there were positive correlations between GR and AOPP (R = 0.934; p < 0.0001) and GR and TG (R = 0.367; p = 0.046). A positive correlation was also revealed between UA and BMI (R = 0.371; p = 0.04). Moreover, serum GSSG concentration was associated with the plasma insulin (R = 0.616; p < 0.0001), HOMA-IR (R = 0.402; p = 0.028) and plasma CRP (R = 0.465; p = 0.006) of the OB subgroup. In OB patients, AOPP was positively correlated with TG (R = 0.402; p = 0.028) (Figure 4).

In the OB + MS subgroup, we showed high positive correlations between GR and AOPP (R = 0.93; p < 0.0001). The positive correlations were also demonstrated between UA and HDL (R = 0.507; p = 0.008) as well as AGE and LDL (R = 0.407; p = 0.031) (Figure 5).

#### 3.2.6. ROC Analysis

**Figure 6:**
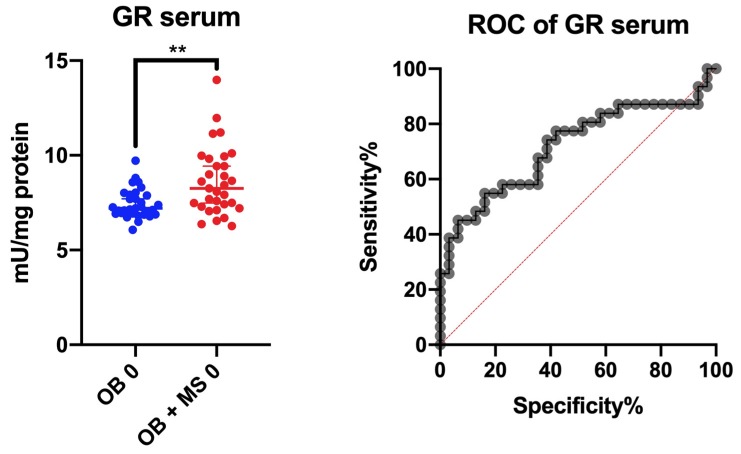
Area under the curve (AUC) of glutathione reductase (GR) activity between the obese patients and cases with obesity and metabolic syndrome. Glutathione reductase (GR), morbid obesity without metabolic syndrome (OB) and morbid obesity with metabolic syndrome (OB + MS). ** p < 0.01 indicate significant difference from the OB 0.

We checked whether the assessed redox biomarkers differentiated cases with morbid obesity from obese cases with metabolic syndrome. Nevertheless, none of the biomarkers (with high specificity/sensitivity) distinguishes the tested groups. The best diagnostic utility has been demonstrated for the serum GR. This parameter with moderate sensitivity (71%) and specificity (61%) differentiates patients with morbid obesity from obese patients with MS (Figure 6). The optimal GR activity differentiating the two groups is >7.450 mU/mg protein at an AUC of 0.72 (p = 0.003).

## 4. Discussion

Numerous studies indicate that visceral obesity is the main cause of insulin resistance and MS [30]; nevertheless, the causes of reduced insulin sensitivity in the target organs are not exactly known. It is suggested that the common denominator of these metabolic disturbances may be redox imbalance as well as increased oxidative stress [31]. Moreover, despite the proven effectiveness of bariatric surgery, it is unclear whether it improves the redox homeostasis of morbidly obese cases. According to our knowledge, this is the first study evaluating the redox balance, glutathione metabolism, and oxidative damage to proteins and lipids in the plasma and serum of morbidly obese patients not only before, but also one, three, six and twelve months after bariatric surgery. We are also the first to compare the redox homeostasis of obese patients with metabolic syndrome to obese cases only.

### 4.1. Antioxidant Barrier

Literature data unequivocally shows that bariatric surgery leads to a marked reduction in adipose tissue mass, followed by an improvement in systemic inflammation [32]. However, with respect to the antioxidant status and oxidative stress, there are some contradictions. Indeed, in obese patients, both increases and decreases in the antioxidant barrier are observed [33,34,35]. These differences may be associated with the duration of obesity as well as the age of obese patients. It is suggested that the antioxidant systems are prompted in the early stage of the disease, whereas in long-term obesity, the source of antioxidants depletes and causes decreased activity of the antioxidant enzymes [34,36]. In our study, the activity of antioxidant enzymes did not differ significantly. Nevertheless, SOD activity was reduced in obese patients both before as well as after the bariatric treatment. In obesity, an important source of ROS is an excessive increase in energetic substrates provided to the respiratory chain in mitochondria. This leads to the formation of considerable amounts of superoxide anions that exceed the antioxidant capacity of the body [37]. However, chronic inflammation is also associated with enhanced production of ROS in obese patients [38,39]. Adipokines secreted by adipose tissue activate the transcription factor NF-κB (nuclear factor-kappa B), which enhances NADPH oxidase (NOX) activity and stimulates phagocytes. Indeed, phagocytes are a rich source of superoxide radicals that produce their large quantities during respiratory burst [39,40]. Under these conditions, the active center of SOD can be inactivated by enhanced levels of free radicals. Although we have not directly evaluated the rate of ROS formation, decreased SOD activity may indicate the long-term overproduction of free radicals in obese patients. Interestingly, Mohseni et al. [41] found a positive correlation between SOD expression and BMI, insulin, HOMA-IR, LDL, total cholesterol and TG. In our study, the activity of SOD does not normalize after bariatric treatment, which suggests the persistence of redox imbalance in obese individuals. Indeed, although body weight has been significantly reduced after the surgery, all patients are still obese and their fat tissue may be an important source of ROS and inflammation.

The antioxidant reserves may also deplete with age. However, our patients were in a similar age range, so the effect of age on the evaluated redox biomarkers is limited [42].

The activity of CAT and GPx did not differ significantly. However, the activity of GR was statistically higher in patients with obesity and MS before bariatric surgery (as compared to controls and obese patients without MS). Considering that GR participates in the regeneration of GSH, an increase in enzyme activity may be an adaptive reaction related to a decrease in glutathione synthesis [24,36]. Indeed, GSH concentration was significantly lower in the plasma of both study groups as compared to healthy controls. Moreover, an increase in GR activity may respond to the overproduction of ROS and the enhanced oxidative damage in the cell. This hypothesis may be confirmed by the strong positive correlation between GR activity and protein oxidation products (AOPP) observed in the study group.

The glutathione level was also significantly reduced in obese patients with MS after the bariatric treatment. This indicates a higher depletion of glutathione reserves in patients with obesity and metabolic syndrome. It should be recalled that GSH is the most important intracellular antioxidant. Reduced glutathione can react with superoxide and hydroxyl radicals, leading to an accumulation of GSSG in the cytosol [43]. However, GSSG can also react with thiol groups of proteins, contributing to the induction of oxidative stress [43]. In our study, GSSG concentration was higher in obese patients with metabolic syndrome before the bariatric treatment (Figure 1B). The positive correlation between GSSG concentration and insulin level/HOMA-IR is also interesting, which may indicate a potential link between glutathione oxidation and disease progression. Importantly, the redox status of obese cases was significantly reduced before the surgery, similarly to obese patients with MS at 3 and 6 months after the surgery. The redox status (ratio of reduced and oxidized glutathione) is used to assess the oxidative-reduction potential of the sample and characterizes the resultant ability of the biological system to counteract oxidative stress [26]. Thus, the oxidant/antioxidant balance is disturbed in patients before the bariatric treatment. However, redox homeostasis is also impaired in obese patients with MS after the surgery. In opposition to our results, Bankoglu et al. [44] found greater GSH and GSSG content in erythrocytes from obese patients before bariatric surgery. Sarosiek et al. [17] observed an increase in oxidized forms of glutathione and cysteine in obese subjects after bariatric surgery.

Uric acid is the end-product of endogenous and dietary purine metabolism [45]. It is also the most important body antioxidant responsible for up to 70%–80% of plasma antioxidant capacity [40]. In low concentrations, UA effectively sweeps oxygen/nitrogen free radicals; nevertheless, at elevated concentrations, it also generates ROS and oxidizes cellular biomolecules [46]. It has been demonstrated that hyperuricemia is associated with obesity, especially with the accumulation of visceral fat [47]. Additionally, evaluated serum UA may imply an increased risk of various metabolic disorders, such as glucose intolerance, T2DM, high blood pressure, metabolic syndrome as well as cardiovascular disease [45,48,49]. Indeed, hyperuricemia is responsible for reducing the release of nitric oxide (NO) and the resulting endothelial dysfunction/platelet aggregation [46]. Although these reactions may be partially blocked by glutathione, when the concentration of GSH decreases, there is an increased production of nitrogen free radicals. However, how does bariatric treatment affect the blood UA levels? Liu et al. [45] observed a decrease in serum UA of women and men one year after bariatric surgery. However, in our study, a statistically significant change was found only in patients from the OB 3 group. No significant decrease of UA concentration after bariatric surgery, especially in the OB + MS group, may be associated with the very high weight of all patients (Figure 1E). Our patients had class 3 obesity (mean BMI > 40 kg/m^2^), and those described by Liu et al. [45] had BMI < 35 kg/m^2^ (class 1 obesity). This confirms our earlier hypothesis about the persistence of redox imbalance after bariatric surgery.

### 4.2. Oxidative Damage to Proteins and Lipids

Although changes in the concentration/activity of antioxidants may indicate a systemic redox imbalance, the evaluation of cellular oxidation products is necessary to demonstrate oxidative stress in the biological system [8]. In this study, we assessed the products of protein (AGE, AOPP) and lipid (4-HNE, 8-isop) plasma oxidation. AGEs are pro-oxidant compounds formed through the non-enzymatic glycation of proteins [50]. Their higher formation is caused mainly by hyperglycemia and, therefore, they are a recognized biomarker of protein carbonyl stress [51]. In our research, AGEs content was significantly increased in both studied groups as compared to the healthy control group. AGEs can bind to receptors on the cell surface and influence several intracellular processes. Indeed, by combining with a specific receptor (RAGE), AGEs increase the production of ROS (through NOX induction), but also enhance the expression of the NF-κB pathway. Under these conditions, other signal routes (MAP-kinases, NJK and p21RAS) can be activated [39,52]. It is suggested that AGEs may play a key role in the development of insulin resistance [39,52]. However, the AGE level was diminished twelve months after bariatric surgery. This is undoubtedly related to the improvement of metabolic status in obese people. Indeed, we observed a decrease in pro-inflammatory parameters such as CRP and WBC. Also, the plasma concentration of glucose and insulin, as well as total cholesterol and LDL diminished in both morbidly obese cases (Table 1). Given the key role of protein glycation in the progression of microvascular lesions, a decrease in AGEs after the surgery may explain the reduced incidence of retinopathy, nephropathy and neuropathy in patients undergoing treatment [52,53]. Indeed, the most common cause of microangiopathy is basement membrane thickening and extracellular matrix hypertrophy [53]. In our patients, the AOPPs concentration also decreased after the bariatric surgery. AOPPs are a family of dityrosine-containing products produced by the reaction of proteins with hypochlorous acid, resulting from myeloperoxidase activity [54]. AOPP appears to be superior to other redox markers due to its early formation, greater stability and longer half-life [55]. Increased accumulation of AOPP has been linked with oxidative stress-related diseases and impaired carbohydrate metabolism (obesity, T2DM as well as metabolic syndrome) [56,57,58]. Krzystek-Korpacka et al. [59] observed a reduction in the plasma AOPP levels after the weight loss caused by lifestyle modification (encompassing physical activity and low caloric diet). Nevertheless, so far, no one has examined the impact of bariatric surgery on AOPP levels in morbidly obese patients. Therefore, we have shown that bariatric surgery not only reduces protein glycation (carbonyl stress; AGE) but also decreases protein oxidation in obese cases (AOPP).

In our study we also evaluated lipid oxidation products: 8-isoprostanes and 4-hydroxynonneal protein adducts. Previously, greater levels of 8-isoP and 4-HNE have been shown in diabetes, atherosclerosis, cardiovascular diseases, and cancers [60,61,62]. However, knowledge of lipid oxidation in the course of bariatric treatment is still limited. In our previous study [7] we evaluated lipid oxidation products in the nonstimulated and stimulated saliva of patients with morbid obesity treated with bariatric surgery. We [7] found a significantly higher concentration of 8-isoP and 4-HNE in nonstimulated and stimulated saliva of obese cases before, as well as six months after the surgery. In this study, the 8-isop concentration was significantly greater in both obese groups before bariatric surgery as well as in every time period after the treatment (as compared to the control). This is not surprising because lipids, especially unsaturated ones, are relatively unstable compounds that are easily oxidized. Although lipid oxidation is a complex process, it occurs particularly when the antioxidant barrier is exhausted [26,40]. Thus, in our patients, changes in glutathione metabolism, reduced SOD activity as well as an increase in uric acid level may be crucial. It has been shown that lipid peroxidation products change the physical properties of cell membranes. Indeed, lipoperoxidation leads to a disturbance of cell membrane asymmetry as well as an inhibition of membrane enzymes and transporter proteins [26,40]. However, in morbidly obese cases, the plasma concentration of 8-isop diminished in three and twelve months after the surgery. This suggests a decrease in lipid peroxidation depending on the time of surgical treatment. Nevertheless, the level of 4-hydroxynonneal protein adducts was increased only in obese patients with metabolic syndrome before the bariatric surgery. Although lipid peroxides can react with proteins and nucleic acids, this occurs only under severe oxidative stress [26,40]. Additionally, of all lipid peroxidation products, 4-HNE is considered the most cytotoxic form of lipid damage [26].

### 4.3. Diagnostic Significance

Recently, there has been a growing interest in the use of redox biomarkers in the diagnosis of various systemic diseases [19,36,63,64,65,66,67,68,69]. This is not surprising because oxidative stress plays a key role not only in the pathogenesis of neurodegenerative diseases/cancers but also many metabolic diseases such as insulin resistance, hypertension, diabetes and metabolic syndrome [40]. We compared whether antioxidants/oxidation products can differentiate between patients with obesity and those with obesity and metabolic syndrome. Although the diagnostic criteria for MS are widely known and routinely used, we have checked whether the assessment of a single biomarker can help to diagnose metabolic syndrome. However, none of the parameters with high sensitivity/specificity differentiates the tested groups. The best diagnostic utility has been demonstrated for the serum GR. Interestingly, GR activity also correlates with TG content in obese cases. However, it is necessary to conduct further studies on a larger population of patients.

### 4.4. Study Limitations

Despite a carefully selected group of patients, our work has certain limitations. We are not able to eliminate the influence of hypotensive/antidiabetic drugs on the assessed redox biomarkers. Besides, the study was conducted exclusively on women. We have previously shown that blood redox homeostasis does not depend on sex in healthy controls [42]; however, it is still unclear whether sex does not affect oxidative stress in obese patients undergoing bariatric treatment. Finally, we evaluated only selected antioxidants and biomarkers of oxidative stress, so we cannot fully conclude on redox homeostasis in obese cases. Although the study should be conducted on a larger population of patients, it should be stressed that for long-term observation, we have included a relatively large number of obese patients cautiously selected for accompanying diseases. Thus, our study is the starting point for further basic and clinical research.

## 5. Conclusions

In obese patients, the antioxidant status is disturbed and protein/lipid oxidation is increased. Although bariatric surgery improves redox homeostasis in obese cases, enzymatic and non-enzymatic antioxidant barriers as well as oxidative stress are not found at the control group level. Although the redox homeostasis disorders seem to be similar in patients with obesity and MS as well as obesity itself, cases with metabolic syndrome showed a continuous decrease in the antioxidant status (GSH, [GSH]^2^/[GSSG]), reduced SOD activity and an increase in UA plasma concentration. Given the persistence of glutathione alterations after the bariatric treatment, antioxidant supplementation should be considered in obese patients with MS. Additionally, further long-term studies are needed on a larger population of obese cases.

## Figures and Tables

**Table 1:** Clinical characteristics of the control, patients with morbid obesity without metabolic syndrome (OB) and patients with morbid obesity and metabolic syndrome (OB + MS). Data given as median (minimum and maximum), * p < 0.05, ** p < 0.01, *** p < 0.001, **** p < 0.0001 indicate significant differences from the control, # p < 0.05, ## p < 0.01, ### p < 0.001, #### p < 0.0001 indicate significant differences from the morbid obesity without metabolic syndrome (OB 0) patients before bariatric surgery; ^ p < 0.05, ^^ p < 0.01 ^^^ p < 0.001 ^^^^ p < 0.0001 indicate significant differences from the morbid obesity with metabolic syndrome (OB + MS 0) patients before bariatric surgery, alanine transaminase (ALT), aspartate transaminase (AST), C-reactive protein (CRP), high-density lipoprotein (HDL), hemoglobin (HGB), homeostatic model assessment of insulin resistance (HOMA-IR), low-density lipoprotein (LDL), platelet count (PLT), red blood cell count (RBC), triacylglycerol (TG), white blood cell count (WBC), waist-hip ratio (WHR), morbidly obese patients without metabolic syndrome (OB) and morbidly obese patients with metabolic syndrome (OB + MS), before (OB 0; OB + MS 0) as well as one month (OB 1; OB + MS 1), three months (OB 3; OB + MS 3), six months (OB 6; OB + MS 6) and twelve months (OB 12; OB + MS 12) after bariatric surgery.

	C	OB 0	OB + MS 0	OB 1	OB + MS 1	OB 3	OB + MS 3	OB 6	OB + MS 6	OB 12	OB + MS 12
**Age**	42 (28–56)	39 (28–52)	49 (28–56)	-	-	-	-	-	-	-	-
**Weight (kg)**	62 (55–72)	118**** (99–169)	125**** (94–170)	107.5**** (90–156)	113.5**** (84–160)	95.5****^##^ (80–124)	102**** (77–138)	85**^####^ (72–113)	92.5****^^^^^^ (70–125)	78^####^ (63–104)	85*^^^^^^ (64–112)
**BMI (kg/m^2^)**	23.04 (21.93–24.80)	44.87**** (40.16–61.34)	46.52**** (40.03–60.96)	40.39**** (30.34–58.01)	41.62**** (32.81–56.68)	36.19****^##^ (28.34–46.86)	39.30**** (29.05–49.91)	32.03**^####^ (26.15–42.06)	34.41****^^^^^^ (26.21–47.62)	28.73^####^ (24.09–37.65)	31.23*^^^^^^ (22.67–40.15)
**WHR**	0.71 (0.64 – 0.74)	0.95**** (0.81–1.04)	0.97**** (0.84–1.15)	0.95**** (0.73–1.01)	0.98**** (0.87–1.15)	0.93**** (0.80–1.01)	0.98**** (0.83–1.12)	0.92*** (0.81–0.98)	0.96**** (0.86–1.13)	0.90*^#^ (0.80–0.98)	0.93***^^^^ (0.84–0.99)
**Waist circumference (cm)**	73 (66–86)	132.5 (110–149)	140.5 (120–161)	125 (100–135)	134.5 (115–152)	112 (90–129)	121.5 (101–139)	101.5 (82–116)	110 (92–130)	92 (78–108)	100 (82–116)
**CRP (mg/L)**	5.5 (5.1–6.5)	8.8 (1.5–27.6)	11.7**** (5.3–18.2)	5.2 (0.5–26)	8.35 (1.5–13.6)	6.1 (0.3–16.5)	7.1 (0.6–16.5)	4.76 (0.3–19.5)	6.05^^^^ (0.5–11.05)	4.85^##^ (0.2–7.8)	5.5^^^^^ (1.2–16.1)
**Glucose (mg/dL)**	76 (67–92)	98**** (76–122)	106*** (89–189)	94**** (69–115)	99**** (75–147)	88 (60–142)	97**** (79–116)	84.5^#^ (68–109)	95**** (81–157)	84^##^ (73–111)	90**^^^^^ (77–99)
**Insulin (uIU/mL)**	7.5 (7–9.4)	17.85**** (8.2–38.6)	22.25*** (9.3–40.7)	11.6**^#^ (4–28)	15.15**** (5.8–35.3)	8.15^####^ (2.7–16.2)	10.5*^^^^^ (3.6–43.8)	8.9^####^ (4.1–12.3)	8.75^^^^^^ (4.5–17.1)	8.1^####^ (3–14.5)	8.5^^^^^^ (4.5–9.9)
**HOMA-IR**	1.44 (1.19–1.86)	4.15**** (1.78–9.34)	5.64*** (3.05–8.1)	2.77***^#^ (0.73–6.56)	3.82**** (1.25–8.62)	1.94^####^ (0.46–3.8)	2.47****^^^^ (0.95–10.16)	1.88^####^ (0.75–2.91)	1.98*^^^^^^ (1.1–3.84)	1.67^####^ (0.58–3.50)	1.91^^^^^^ (1.07–2.17)
**ALT (IU/L)**	23 (16–35)	24 (6–93)	27 (12–54)	23.5 (6–52)	27.5 (14–56)	20 (6–43)	24 (13–59)	17 (6–51)	21 (12–31)	18 (8–37)	19 (8–43)
**AST (IU/L)**	22 (16–36)	19 (13–44)	21 (14–50)	23.5 (14–98)	27 (14–85)	19 (12–36)	21 (13–43)	17 (9–48)	20 (12–43)	18 (12–52)	19 (12–37.2)
**Cholesterol (mg/dL)**	175 (159–189)	195 (147–231)	211**** (167–268)	170.5 (124–210)	192* (126–235)	176 (120–217)	181^^^ (120–237)	178 (114–245)	188 (148–264)	176 (114–231)	174^^^^^ (138–295)
**LDL (mg/dL)**	119 (115–123)	124.5 (100–159)	144** (122–181)	110 (83–153)	122^^^^ (66–183)	106 (69–159)	116^^^^^ (50–189)	109 (61–166)	110^^^^^^ (83–157)	113^#^ (59–134)	102^^^^^^ (64–164)
**TG (mg/dL)**	135 (119–145)	128.5 (62–197)	150 (104–289)	108.5 (66–209)	136 (75–367)	103.5** (46–182)	137 (78–240)	96****^##^ (48–182)	130 (89–214)	98****^##^ (36–182)	102**^^^^^^ (59–146)
**HDL (mg/dL)**	60 (45–68)	50** (33–62)	46**** (31–72)	47**** (30–106)	45**** (31–68)	47*** (27–141)	49*** (35–69)	50** (35–72)	52.5 (35–86)	53 (38–81)	56^^^^ (43–70)
**WBC (10^3^/μL)**	7.5 (4.4–9.1)	8.295* (5.26–12.15)	9.53* (5.85–12.91)	6.53^##^ (4.63–9.64)	7.16 (4.95–12.7)	5.78*^####^ (4.61–10.54)	6.9 (3.9–11.54)	5.98^###^ (4.75–9.27)	7.195 (4.4–10.94)	5.92*^####^ (4.07–8.52)	6.96^^^^^ (4.21–10.26)
**RBC (10^6^/μL)**	4.6 (3.9–5.2)	4.7 (3.51–5.78)	4.6 (4–5.46)	4.79 (4.11–6.03)	4.77 (4.31–6.01)	4.655 (4.11–5.13)	4.75 (4.11–5.45)	4.65 (3.98–5.69)	4.75 (4.02–5.66)	4.62 (4.03–5.4)	4.50 (3.33–5.65)
**HGB (g/dL)**	13.8 (11.5–14.7)	13.1 (11–15.2)	13.35 (12.1–16.2)	13.2 (10–15.9)	13.6 (11.2–16.6)	13.1 (11.5–15.7)	13.2 (10.9–16.3)	13.4 (9.7–15.4)	13.4 (10.6–16.3)	13.65 (9.1–15.8)	14.15 (8.7–16.2)
**PLT (10^3^/μL)**	289 (265–315)	264.5 (141–417	258 (183–418)	261 (121–412)	229* (128–405)	265.5 (130–312)	247 (131–345)	273.5 (188–425)	251.5 (167–375)	214.5*** (130–345)	224** (164–378)

## Supplementary Materials

The following are available online at https://www.mdpi.com/2077-0383/9/4/976/s1, Table S1: Individual data of metabolic parameters in the study group.
